# 

**Published:** 1892-06

**Authors:** 


*The following donations have been received since the publication of the List in March:*

*May* 31*st*, 1892.

| | | |
|---|---|---|
| J. Michell Clarke, M.B. (1) ... ... ... ... | 3 | volumes. |
| C. F. Pickering. (2) ... ... ... ... ... ... ... | 11 | „ |
| Arthur B. Prowse, M.D. (3) ... ... ... ... | 1 | volume. |
| The Staff of St. George's Hospital. (4) ... | 10 | volumes. |
| J. Greig Smith. (5) ... ... ... ... ... ... ... | 16 | „ |
| R. Shingleton Smith, M.D. (6) ... ... ... | 12 | „ |
| Simeon Snell. (7) ... ... ... ... ... ... ... | 1 | volume. |
| P. Watson Williams, M.D. (8) ... ... ... | 2 | volumes. |

Unbound periodicals have been received from Dr. Michell Clarke, Mr. L. M. Griffiths, Mr. W. H. Harsant, and Mr. F. P. Lansdown.

Framed pictures have been presented by Mr. E. J. Burder and Dr. W. H. Spencer.

### FOURTH LIST OF BOOKS.

The figures in brackets refer to the figures after the names of the donors, and show by whom the volumes were presented. The books to which no such figures are attached have either been bought from the Library Fund or received through the *Journal.*

---

**Acton, W.** ... ... *Prostitution* ... ... ... ... ... ... ... ... ... ... (2) 1857
**Adams, W.** ... ... *On Finger-Contractions and Hammer-Toe.* 2nd Ed. 1892
**Aitken, W.** ... ... *The Science and Practice of Medicine.*
(1) Vol. I. 3rd Ed. 1864

**Ball, M. V.** ... ... *Essentials of Bacteriology* ... ... ... ... ... ... ... 1891

**Barton, J. K.** ... *Tables for the Diagnosis and Treatment of Syphilis.* 3rd Ed. [1892]
**Boyce, R.** ... ... *A Text-book of Morbid Histology* ... ... ... ... 1892
**Bramwell, B.** ... *Atlas of Clinical Medicine.* Vol. I., Part IV. ... 1892
**Budd, G.** ... ... *Diseases of the Liver* ... ... ... ... (2) 3rd Ed. 1857

**Carrington, R. E.** *Notes on Pathology* ... ... ... ... ... ... ... ... 1892
*Catalogue (Encyclopædic) of the Guille-Allès Library and Museum, Guernsey* 1891
**Curgenven, J. B.** *The Disinfection of Scarlet Fever* ... ... ... ... 1891

**Davis, H.** ... ... *Anæsthetics* ... ... ... ... ... ... ... ... 2nd Ed. 1892

**Eisenberg, J.** ... *Bacteriological Diagnosis* ... ... ... ... 2nd Ed. 1892

**Fagge and P. H. Pye-Smith, C. H.** *Text-Book of the Principles and Practice of Medicine.* 2 vols. ... (8) 3rd Ed. 1891

**Gemmel, W.** ... *Dermic Memoranda* ... ... ... ... ... ... ... [1892]
**Goodhart, J. F.** .. *On Common Neuroses* ... ... ... ... ... ... ... ... 1892
**Gray, H.** ... ... *Anatomy* ... ... ... ... ... ... ... ... (6) 1858

**Hope, J.** ... ... *On the Diseases of the Heart* ... ... (2) 3rd Ed. 1839
**Howard, F.** ... *On the Teeth* ... ... ... ... ... ... (2) 40th Ed. 1857
**Hughes, H. M.** ... *Practice of Auscultation* ... ... ... (2) 2nd Ed. 1854

**Jones and E. H. Sieveking, C. H.** *Pathological Anatomy* ... ... (2) 1854

**Kingsbury, G. C.** *The Practice of Hypnotic Suggestion* ... ... (5) 1891

**Lane, H.** ... ... *Differentiation in Rheumatic Diseases (so-called).* 2nd Ed. 1892
**Lawrance, D. D. Stewart and E. S.** *Essentials of Medical Electricity* ... 1892
**Lewers, A. H. N.** *Diseases of Women* ... ... ... ... (5) 2nd Ed. 1890
**Lowndes, F. W.** ... *Reasons why the Office of Coroner should be held by a Member of the Medical Profession* ... ... ... [N.D.]

**Neale, R.** ... ... *The Medical Digest* ... ... ... ... ... ... ... (6) 1877

**Ormerod, J. A.** ... *Diseases of the Nervous System* ... ... ... ... ... ... 1892
**Osler, W.** ... ... *The Principles and Practice of Medicine* ... ... ... 1892

**Page, H. W.** ... *Railway Injuries* ... ... ... ... ... ... ... ... (5) 1891
**Pye, W.** ... ... *Surgical Handicraft.* 3rd Ed. (Ed. by T. H. R. Crowle) ... ... ... ... ... ... ... ... (5) 1891
**Pye-Smith, C. H. Fagge and P. H.** *Text-Book of the Principles and Practice of Medicine.* 2 vols. ... (8) 3rd Ed. 1891

**Quain, J.** ... ... *Anatomy.* 10th Ed. (Ed. by E. A. Schäfer and G. D. Thane). Vol. II., Part II. ... ... ... 1892

**Rideal, S.** ... .. *Practical Chemistry* ... ... ... ... ... ... (5) 1890

**Robson, A. W. M.** *On Gall-Stones* ... .. ... ... ... ... ... ... 1892

**Rosenthal, M.** ... *Elektrotherapie* ... ... ... ... ... ... ... (6) 1873

**Schweinitz, G. E. de** *Diseases of the Eye* ... ... ... ... ... ... ... 1892
**Shaw, J.** ... ... *Epitome of Mental Diseases* ... ... ... ... (5) 1892
**Sheen, A.** ... ... *Workhouse Medical Officer* ... ... (5) 2nd Ed. 1890
**Sieveking, C. H. Jones and E. H.** *Pathological Anatomy* ... ... (2) 1854
**Snell, S.** ... ... *A History of the Medical Societies of Sheffield* (7) 1890
**Stewart and E. S. Lawrance, D. D.** *Essentials of Medical Electricity*... 1892

**Todd, R. B.** ... *Clinical Lectures—Nervous System* ... (2) 2nd Ed. 1856
,, ... ... ,, *Urinary Diseases*... ... ... (2) 1857

**Treves, F.** ... ... *The Student's Handbook of Surgical Operations* ... 1892

**West, C.** ... ... *On Diseases of Women.* Two Parts ... (2) 1856-58
**White, W. H.** ... *Materia Medica* ... ... ... ... ... ... ... ... ... 1892
**Williams, C. J. B.** *Principles of Medicine* ... ... ... (2) 3rd Ed. 1856

### TRANSACTIONS, REPORTS, JOURNALS, &c.

*The List of current Periodicals to be seen in the Library is given in the Journal for March.*

Alienist and Neurologist, The Vol. XII. ... ... ... ... ... ... 1891
American Practitioner and News, The Vols. XI. and XII. ... ... 1891
American Surgical Association, Transactions of the Vol. IX. ... 1891
Annals of Surgery ... ... ... ... ... ... ... ... ... (5) Vol. X. 1889
Australian Medical Journal, The New Series ... ... (5) Vol. X. 1888
*Bäder-Almanach* ... ... ... .. ... ... ... ... ... ... ... (3) [1892]
Birmingham Medical Review, The... ... ... ... (5) Vol. XXV. 1889
Bulletin de l'Académie de Médecine Tomes XXV. & XXVI. ... 1891
Canada Medical and Surgical Journal ... ... ... (5) Vol. XVI. 1888
Cincinnati Lancet and Clinic, The ... ... ... ... ...(5) Vol. LII. 1884
(Continued as *The Cincinnati Lancet-Clinic.*)

Cincinnati Lancet-Clinic, The Vol. LXV. ... ... ... ... ... ... ... ... 1891
(Continuation of *The Cincinnati Lancet and Clinic.*)
Dublin Journal of Medical Science, The (6) Vols. LXXVII.—LXXX., 1884-85; (5) Vol. LXXXVIII., 1889; Vols. XCI., XCII. 1891
Edinburgh Medical Journal (5) Part II., Vol. XXXIV., 1889; Part II., Vol. XXXVI., Part I., Vol. XXXVII. 1891
Epitome of Medicine, The Vol. VIII. ... ... ... ... ... ... ... ... 1891
(Continuation of *The Medical Analectic and Epitome.*)
Fortschritte der Krankenpflege ... ... ... ... ... ... ... ... ... 1891
Gazette de Gynécologie Tome VI. ... ... ... ... ... ... ... ... 1891
Gazette des Hôpitaux .. ... ... ... ... ... ... ... ... ... ... ... 1891
Hospital, The Vol. XI. ... ... ... ... ... ... ... ... ... ... 1892
Hospitals—
Saint Bartholomew's Hospital Reports (6) Vols. VIII., IX., XII., XIII. 1872-77
St. George's Hospital Reports (4) Vols. I.—VI., 1866-73; (4, 6) Vol. VII., 1875; (4) Vols. VIII.—IX. 1877-80
Index Medicus Vol. XIII. ... ... ... ... ... ... ... ... ... 1891
International Clinics Vols. III., IV. ... ... ... ... ... ... ... ... 1892
Journal of Anatomy and Physiology, The (1) Vols. XX., XXI. 1886-87
Journal of Laryngology and Rhinology, The Vol. V. ... ... ... 1891
Medical and Surgical Reporter, The .. ... ... (5) Vol. LVI. 1887
Medical Annual, The ... ... ... ... ... ... ... ... ... ... ... 1892
Medical News, The Vol. LIX. ... ... ... ... ... .. ... ... 1891
Progrès Médical, Le Tome XIV. ... ... ... ... ... ... ... ... 1891
Revue de Laryngologie, d'Otologie et de Rhinologie Tome XI. ... 1891
Royal Academy of Medicine in Ireland, Transactions of the Vol. IX. 1891
St. Louis Medical and Surgical Journal, The ... (5) Vol. LVII. 1889
Year-Book of Scientific and Learned Societies ... ... ... ... ... 1892

